# Assessing frailty in elderly patients with hip fractures: A retrospective review comparing geriatrician and orthopedic trainee assessments

**DOI:** 10.1097/MD.0000000000036336

**Published:** 2023-11-24

**Authors:** Kit Moran, Matthew J. Laaper, Emma E. Jones, Chad P. Coles, William M. Oxner, Paige A. Moorhouse, R. Andrew Glennie

**Affiliations:** a Department of Surgery, Dalhousie University, Halifax, Nova Scotia, Canada; b Faculty of Medicine of Memorial University, St. Johns, Newfoundland, Canada; c Nova Scotia Health, Halifax, Nova Scotia, Canada; d Division of Geriatric Medicine, Dalhousie University, Halifax, Nova Scotia, Canada.

**Keywords:** CFS, frailty, hip fracture, orthopedic

## Abstract

To assess the correlation of orthopedic surgery residents compared with expert geriatricians in the assessment of frailty stage using the Clinical Frailty Scale (CFS) in patients with hip fractures. A retrospective chart review was performed from January 1, 2015 to December 31, 2019. Patients admitted with a diagnosis of hip fracture were identified. Those patients with a CFS score completed by orthopedic residents with subsequent CFS score completed by a geriatrician during their admission were extracted. Six hundred and forty-eight patients over age 60 (mean 80.5 years, 73.5% female) were admitted during the study period. Orthopaedic residents completed 286 assessments in 44% of admissions. Geriatric medicine consultation was available for 215 patients such that 93 patients were assessed by both teams. Paired CFS data were extracted from the charts and tested for agreement between the 2 groups of raters. CFS assessments by orthopedic residents and geriatrician experts were significantly different at *P* < .05; orthopedic residents typically assessed patients to be one CFS grade less frail than geriatricians. Despite this, the CFS assessments showed good agreement between residents and geriatricians. Orthopaedic surgery residents are reliable assessors of frailty but tend to underestimate frailty level compared with specialist geriatricians. Given the evidence to support models such as orthogeriatrics to improve outcomes for frail patients, our findings suggest that orthopedic residents may be well positioned to identify patients who could benefit from such early interventions. Our findings also support recent evidence that frailty assessments by orthopedic surgeons may have predictive validity. Low rates of initial frailty assessment by orthopedic residents suggests that further work is required to integrate more global comprehensive care.

## 1. Introduction

Hip fractures are a common, prognostically significant injury in older adults. Patients with hip fractures are at risk of morbidity, extended hospital stays, inability to return to previous level of function, and mortality.^[1–6]^ Some authors have explored the predictive validity of frailty for poor health outcomes in hip fracture patients^[3–7]^ and recent efforts have been made to identify and intervene in those most at risk using a frailty framework.^[2–6,8–13]^

The Clinical Frailty Scale (CFS) is one of many validated frailty identification tools.^[12–17]^ Based on longitudinal data from the Canadian Study on Health and Aging, the CFS provides a scale for evaluating a patient on the continuum of frailty. It classifies patients on a 9 point ordinal scale, with scores 1 to 3 indicating non-frail patients, and scores of 4 or greater indicating frailty.^[15]^ It has been validated as a predictive measure of adverse health outcomes in general geriatric populations.^[16]^

The CFS been shown to be easy-to-use with good inter-rater reliability in untrained general medical trainees.^[14,18–20]^ These features are critical if frailty levels, particularly frailty level cutoffs are to be used for clinical decision making. This issue has been well-studied in the cardiology literature, in part because of the incorporation of frailty in heart transplantation screening.^[21]^ One study demonstrates that the unstructured “foot-of-the-bed” or “eyeball” tests (i.e., clinical gestalt) can lack reproducibility in assessing frailty.^[21–27]^ Several authors have suggested the CFS framework is a superior replacement for unstructured foot-of-the-bed assessments.^[27–30]^ It provides a structure to summarize and communicate frailty as assessed from a clinical encounter.^[31]^

In the orthopedic literature, attention to frailty has been growing although there is limited consensus on a common approach to responding to frailty when it is identified. Two recent studies have shown the predictive validity of the CFS for adverse outcomes after hip fracture surgery.^[3,4]^ Despite this, many orthopedic surgeons still have little recognition or understanding of frailty in their patients beyond its use as a general stratification of risk.^[7]^ Lack of frailty literacy presents a significant dilemma: how should orthopedic surgeons incorporate frailty into clinical decision-making given limited working knowledge of frailty?^[32]^ This will become of particular issue if one or more frailty thresholds are identified which significantly impact patient care or clinical decision-making.^[8]^

Although the CFS has previously been shown to have good inter-rater reliability in untrained medical trainees, this has not been replicated in orthopedic surgery trainees.^[14]^ There is a paucity of literature comparing how well surgeons or surgical trainees identify and assess frailty, especially in direct comparison to expert geriatricians.^[32–34]^ Our study seeks to answer how often orthopedic surgery residents formally assess frailty in hip fracture patients and how closely these evaluations correlate with those performed by expert geriatrician assessors.

## 2. Materials and Methods

### 2.1. Current hip fracture pathway

At the tertiary academic health center, elderly patients with a diagnosis of low-energy hip fracture are admitted to a defined clinical pathway with standardized documentation. Patients were followed on the acute care ward and the rehabilitation ward connected to the main building. Upon diagnosis of hip fracture by an emergency department physician, the orthopedic service is consulted. The orthopedic resident documents the CFS score on the hip fracture pathway forms. Although there is a dedicated section on the standardized form, completion of the CFS score is not mandatory for admission. The resident received no orientation or introduction to using or assessing patients with the CFS.

Geriatric Medicine consultation services can be initiated by orthopedics during pre-operative, peri- operative, post-operative, or post-acute care rehabilitation for assessment of frailty, medical issues (including delirium), mobility and home support planning, and medical/surgical decision-making. Institutional practice for Geriatric Medicine consultation is to complete a Comprehensive Geriatric Assessment which includes a detailed assessment of frailty and function. A summary classification of frailty is then categorized on the CFS. The nature of the CFS itself accounts for a number of potential confounders. During ethics review it was noted that many of the baseline demographic variables were proposed to collect were rejected given the information that was more pertinent and available within the scale itself.

### 2.2. Study design and patients selection

This study is a retrospective cohort study approved by the Nova Scotia Health Research Ethics Board. A chart review was performed using a convenience sample of paired CFS data for hip fractures at a single time-point (a single inpatient admission). The nature of a retrospective review is highly biased (level 4). We analyzed scores that were collected prospectively but then analyzed them retrospectively. The scores were not altered in any way and there was broad representation from both the orthopedics division and the division of geriatric medicine, that is, >10 in each group. To accomplish this, the authors reviewed medical records from January 1, 2015 to December 31, 2019 to identify patients with an admitting diagnosis of hip fracture (ICD-10 - S72.0, S72.1 or S72.2).

Patients less than 60 years of age were excluded, as this is the generally accepted lower-limit for fragility-type hip fractures. Below this age, hip fractures are felt to represent other mechanisms than frailty, which is outside the primary focus of this study.

Only patients undergoing surgery were included in the patient sample. Non-surgical hip fractures were excluded as they are typically a palliative scenario at our institution and would not be seen by either orthopedics or geriatric teams. Patients that had documented assessments on the CFS by both the orthopedic and geriatric teams were included in the study. Patients were excluded if one or both CFS assessments were not performed.

### 2.3. Statistical analysis

Statistical analysis was performed with R version 4.2.1: RStudio Inc. (2022-06-23). Tests included Fisher’s exact test for categorical variables of demographic data, Wilcoxon signed rank for paired difference in CFS, and linear and Bland-Altman tests for visual inspection of agreement.

For demographic data, patients were also analyzed in subsets of the CFS in keeping with previous studies.^[4,15,19,20,30]^ Those with scores 1 to 3 were classified as “non-frail”; CFS scores above 4 were classified as frail, with a further subset of frail patients with scores 7 to 9 classified as “severely frail.”

Quantitative assessment of agreement of paired CFS data was calculated by percentage agreement, but this does not account for the influence of agreement arising from chance. Thus, Gwet’s agreement coefficient from R package irrCRC (K Gwet, 2019, version 1)^[35]^ was also used. This test has tolerance for multiple unknown raters and can be used on ordinal data in uneven distributions.^[35–39]^ Using Gwet’s agreement coefficient, analysis was completed using both unweighted (AC1) and weighted (AC2) scores. Weighting was done in an ordinal weighting scheme, reflecting of number of CFS categories.^[36,39,40]^ Given that the literature shows no defined transition points or clinical decision cutoffs for frailty in this population, we propose that “close-but-not-perfect” agreement was acceptable in this application of the CFS scale.^[14,41]^ Statistical significance was set at a *P* value of less than .05.

Independent statistical support was sought from the local Research Methods Unit. Full data was shared to assess the appropriateness of the statistical tools used in the analysis.

## 3. Results

### 3.1. Patient demographic data

**Figure 1.**
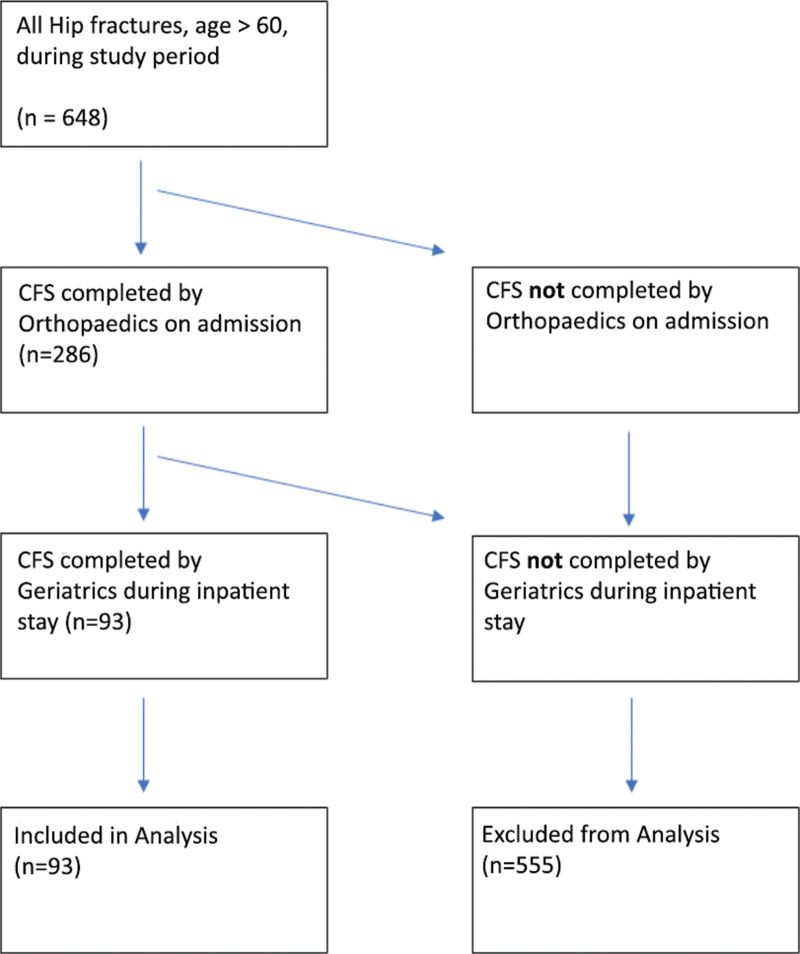
Fates of all participants.

Six hundred forty-eight patients over the age of 60 (mean 80.5 years, 73.5% female) were admitted between 1/1/2016 and 7/6/2021 were included in the initial evaluation. Of those, 286 patients (44%) had CFS assessment by orthopedics and 215 patients (33%) had CFS assessments by geriatrics. Ninety-three patients (14.4%) received both an orthopedic and a geriatric assessment and were included in the study (Fig. 1).

**Figure 2.**
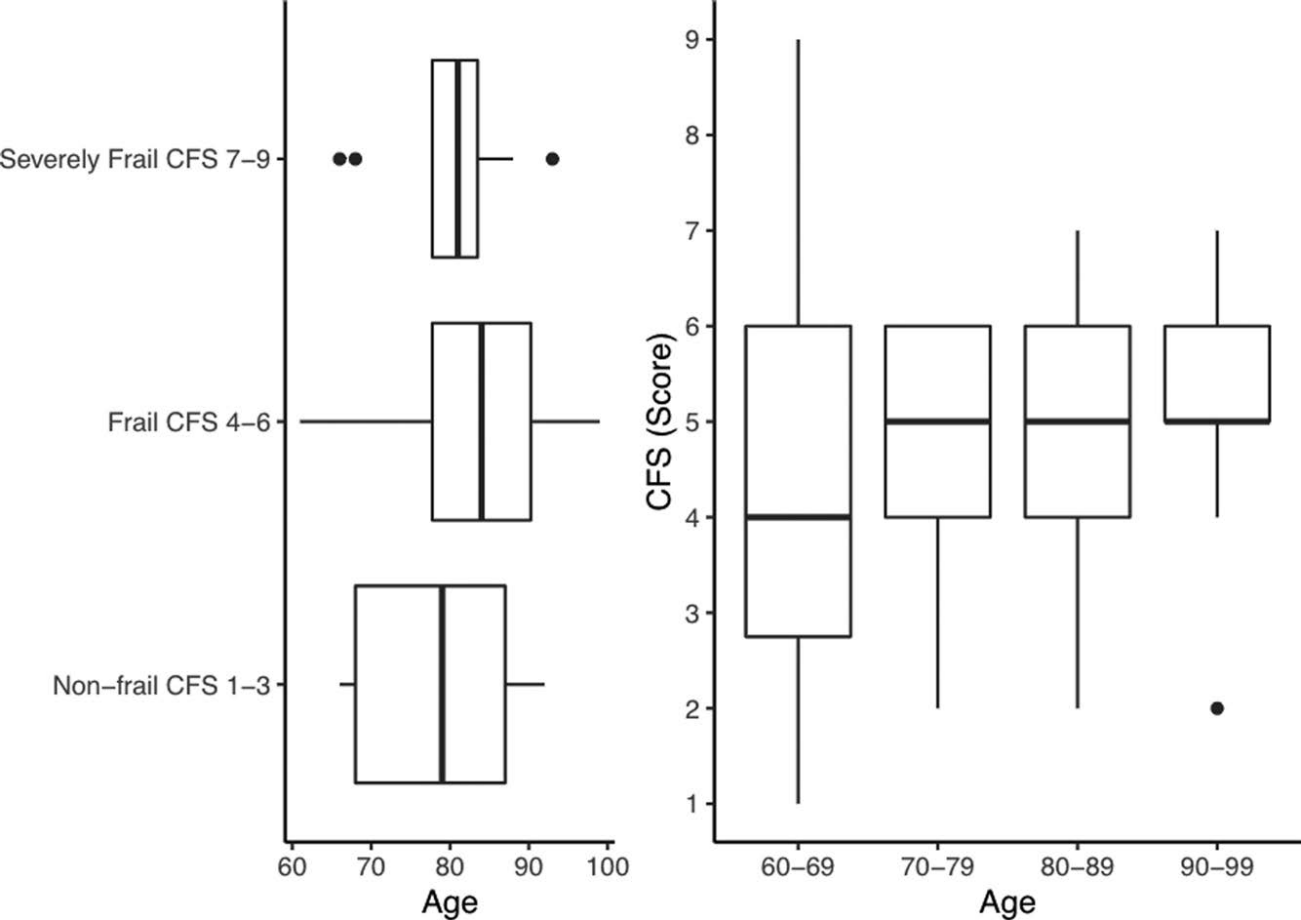
Distribution of age and frailty.

**Figure 3.**
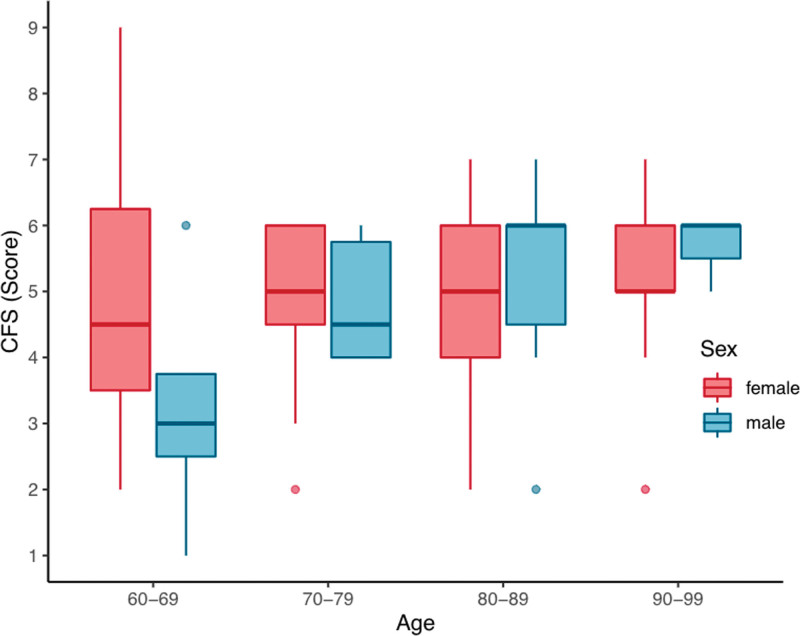
Distribution of age and frailty by sex.

Descriptive data stratified by frailty classification are shown in Table 1. Chronological age alone did not explain frailty level but cohorts did tend to be frailer with advancing age (*P* = .049) as seen in Figure 2. There was no significant difference in frailty on the basis of sex alone (*P* = .623, Fig. 3).

**Table 1:** Patient demographic data and frailty status.

Characteristics		Non-frail CFS 1–3	Frail CFS 4–6	Severely Frail CFS 7–9	*P*
Age	Mean (SD)	78.5 (9.5)	83.4 (8.5)	80.0 (9.1)	.098
Cohort	60–69	5 (29.4)	5 (7.4)	2 (25.0)	.049*
	70–79	4 (23.5)	21 (30.9)	0 (0.0)	
	80–89	7(41.2)	25 (36.8)	5 (62.5)	
	90–99	1 (5.9)	17 (25.0)	1 (12.5)	
Sex	Female	13 (76.5)	49 (72.1)	7 (87.5)	.623
	Male	4 (23.5)	19 (27.9)	1 (12.5)	

Fisher’s exact test for significance, where * denotes significance at *P* < .05 level. Cohort reported as sample size by frailty level (n) with percentage in brackets (%).

### 3.2. Clinical Frailty Scores

**Figure 4.**
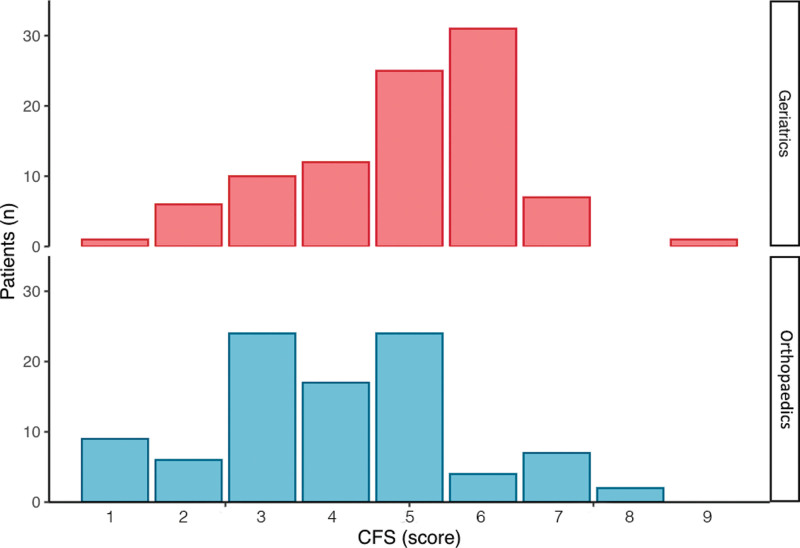
Distribution of frailty by assessing service.

**Figure 5.**
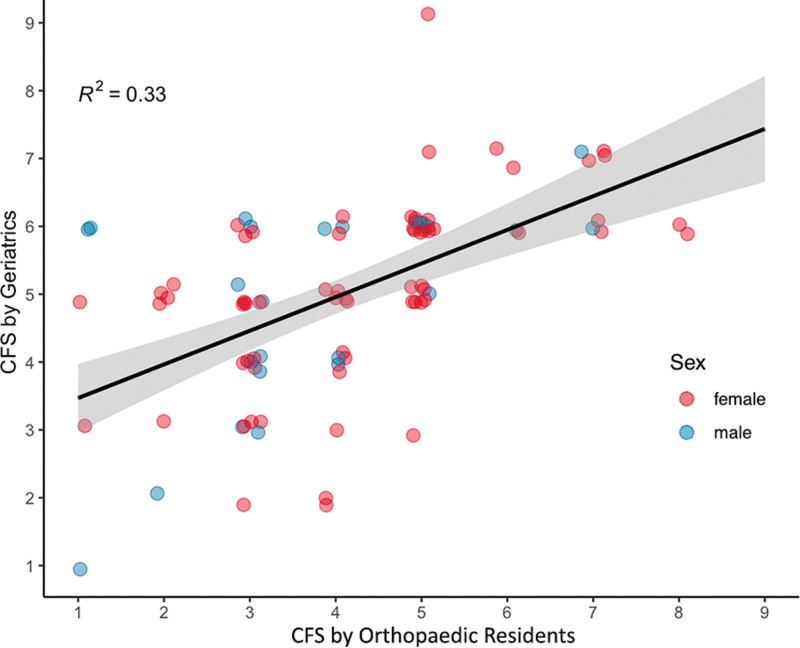
Regression of CFS scores by assessing service. CFS = Clinical Frailty Scale.

**Figure 6.**
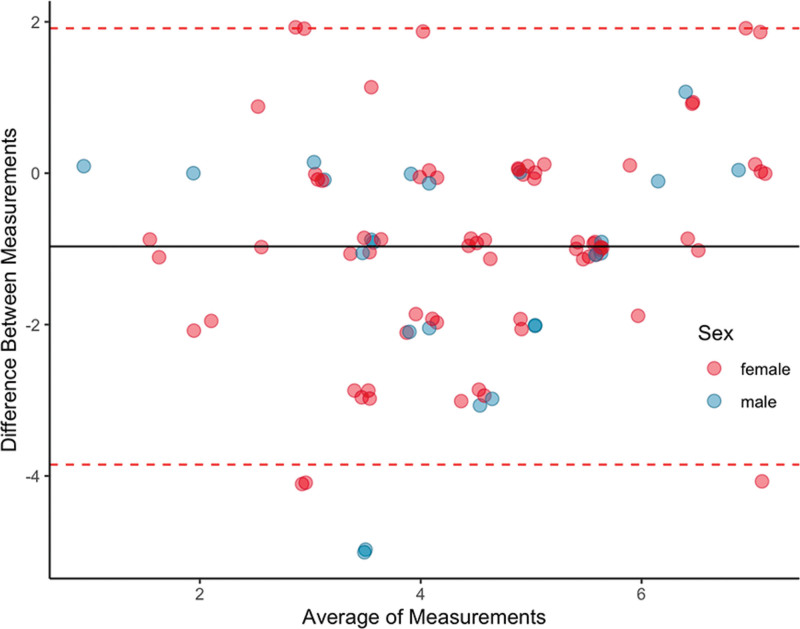
Bland Altman plot of CFS scores. CFS = Clinical Frailty Scale.

Figure 4 shows the distribution of CFS scores by Geriatric and Orthopedics raters. Orthopedic assessments tended to have lower frailty scores, which was confirmed on Wilcoxon signed-rank test (est. 1.50, 95% CI 1.00–1.50, *P* < .05). Linear regression (*r*^2^ = 0.33, *P* < .05, Fig. 5) and Bland-Altman (mean of difference = −0.97, 95% CI 1.91 to −3.85, Fig. 6) showed clustering and central tendency around the mean, despite an agreement difference of approximately 1 frailty level.

The unknown number of raters and uneven distribution of scores (kappa paradox) in an ordinal dataset precluded the use of Cohen’s kappa, which is probably the most well-known test for agreement.^[34–36]^ We therefore used Gwet’s agreement coefficient as a validated method to address these challenges.^[34,40,41]^ The unweighted agreement was 0.20 (95% CI 0.09–0.30, *P* < .05, Table 2) which reflected the low levels of exact agreement (27.96%). This is expected given the known discrepancy between raters. However, with ordinal weighting to recognize close agreement, the agreement coefficient (AC2) is 0.79 (95% CI 0.72–0.85, *P* < .05).^[14,36,40]^ Similar results were found for subsets of patients based on Geriatric assessor assigned frailty scores, with good agreement for both non-frail (0.69, 95% CI 0.47–0.92, *P* < .05) and frail patients (0.78, 95% CI 0.71–0.86, *P* < .05). For the severely frail, small sample size (n = 8) and few possible ratings limited analysis.

**Table 2:** Agreement coefficients with 95% confidence intervals for weighted and unweighted tests.

Data	Weighting	All patients	Non-frail (CFS 1–3)	CFS ≥ 4	CFS ≥ 7
Initial	Unweighted (AC1)	0.20 (0.09–0.30)*	0.29 (−0.04 to 0.62)	0.17 (0.06–0.28)*	0.40 (−0.21 to 1)
	Ordinal weighting (AC2)	0.79 (0.72–0.85)*	0.69 (0.47–0.92)*	0.78 (0.71–0.86)*	0.58 (−0.20 to 1)
Transformed	Unweighted (AC1)	0.25 (0.14–0.35)*	0.04 (−0.20 to 0.28)	0.28 (0.15–0.40)*	0.06 (−0.49 to 61)
	Ordinal weighting (AC2)	0.85 (0.80–0.89)*	0.63 (0.38–0.88)*	0.84 (0.78–0.90)*	0.49 (−0.35 to 1)

* Significant at *P* < .05 level.

Noting the approximate 1 score discrepancy between Geriatric and Orthopedic assessments, a transformation was applied to the orthopedic scores to shift them. The same agreement statistics were then performed again on the transformed data. Overall, similar patterns were seen as with the initial dataset prior to transformation (Table 2).

Neither the initial nor transformed dataset show an association between unweighted ratings by Geriatric and Orthopedic assessors for non-frail patients. Under ordinal weighting, there is moderate agreement, suggesting that there is more variance within this subset. This is supported by visual inspection of the linear regression (Fig. 5) where a substantial portion of points would lie above a line with slope of 1 for CFS < 4. This would persist even after accounting for a 1 class discrepancy in ratings.

## 4. Discussion

The role of frailty assessment as part of an orthopedic assessment is important both for prognostication and shared surgical decision making. In recent studies of similar populations of elderly patients with hip fracture, Ikram et al^[3]^ found an overall significant correlation between higher CFS scores and adverse outcomes, complications, length of stay (LOS), institutionalization, and death. This was supported by Narula et al^[4]^ who also found significant correlation between frailty, LOS, discharge destination and 30-day mortality. The prospects of institutionalization and mortality following hip fracture may be key determinants as to whether patients decide to undergo surgical management.

Interestingly, however, Ikram et al noted that for all but institutionalization, the correlation breaks down with increased frailty (CFS ≥ 4). They observed an initial linear rise of frailty with 30-day mortality before plateauing at CFS > 5. Similarly, the linear rise and plateau was also observed with LOS and rate of complications in contrast to the trend observed by Narula et al where linear increases in frailty were associated with rising 30-day mortality. From this, Ikram et al concluded the admitting orthopedic team does not appear successful in distinguishing between higher CFS categories, perhaps due to lack of expertise.^[3]^

Our results, which are consistent with Narula et al, suggest that orthopedic assessors can provide accurate assessments of frailty for a broad spectrum of patients, and CFS ≥ 4. Although exact agreement was low, there was good weighted-agreement overall between orthopedic residents and expert geriatricians. One caveat is that for severely frail patients (CFS ≥ 7), our study did not have sufficient numbers to draw conclusions about associations.

While orthopedic residents produced lower ratings of frailty compared to expert geriatricians, under-estimating the frailty by 1 grade may be clinically meaningful in some instances.^[14]^ Theo et al argued that their modified form of the CFS that under-called frailty by a single CFS was likely acceptable, except in the case of a “go/no go” threshold. This concept arises in the literature around resource-limited scenarios, where frailty above a threshold value can be a factor in withholding care such as in cardiac procedures and transplantation, or COVID triage.^[21,28,42,43]^ Certainly, from the frailty literature in orthopedics at this time, such a hard discriminator does not exist. We propose that the agreement observed in our study is likely acceptable, and having a frailty assessment performed by orthopedic residents at the time of admission may be the most important step to facilitate earlier intervention and improve outcomes. However, this should be taken with caution, as for some patients with severe frailty, such as those with severe dementia or dependence for basic activities of daily living, undervaluing their frailty could be particularly significant.

Furthermore, the difference in scoring between orthopedic assessors and geriatric assessors could be, in part or in whole, due to the study design. The orthopedic assessments were drawn from the CFS scores assigned in the admission documents, and thus were all completed at the time of admission. The geriatric assessments, on the other hand, were not done at a defined time-point but rather were completed at any time during the inpatient stay. The institutional experience of the authors is that this typically, but not exclusively, would take place after the surgery. This represents a significant confounder to our data, as the discrepancy in paired assessments could possibly be explained by a significant stressor in the assessment interval. Although frailty is a baseline characteristic and should be scored based on functional status prior to the health crisis, the influence of the intervening stressor on frailty scores cannot be excluded in this study design.

The results of this study should be interpreted with caution. As this is a single institution study, with relatively few orthopedic residents generating initial frailty assessments, the findings may not be generalizable or externally valid. Further, the distribution of the frailty scores limits the ability to draw conclusions on the margins. Finally, our statistical analysis of agreement using Gwet’s method may be unfamiliar to some readers but addresses the challenges posed by the unknown number of raters and the uneven distribution of CFS values.

Regardless of under calling frailty levels, surgical residents are well-positioned to perform screening at first contact on admission particularly if they have training in what clinical information to use in their scoring. Their unique position to initiate early frailty screening could be leveraged to facilitate earlier involvement by geriatric specialists, in line with recent efforts to intervene in vulnerable patients to improve outcomes.^[2,8,10,12,32,34]^ Furthermore, the use of scoring aids such as a classification tree could potentially improve the accuracy of untrained assessors and quality of information gathered early in admission.^[14]^ Future studies should be focused on improving the uptake of frailty screening among orthopedic residents; improving the quality of the data used for scoring; and expanding the CFS screening beyond hip fracture patients.

## Acknowledgements

Our statistics were reviewed with the research methods unit (RMU) biostatistics subdivision at the Nova Scotia Health Authority (NSHA).

## Author contributions

**Conceptualization:** Chad Coles, William Oxner.

**Data curation:** Kit Moran, Matthew J. Laaper.

**Formal analysis:** Kit Moran.

**Investigation:** Chad Coles, William Oxner.

**Methodology:** Kit Moran, Matthew J. Laaper.

**Project administration:** Chad Coles, William Oxner.

**Resources:** Kit Moran, Matthew J. Laaper.

**Software:** Kit Moran.

**Supervision:** Paige Moorhouse, Andrew Glennie.

**Visualization:** Emma Jones.

**Writing – original draft:** Kit Moran, Emma Jones.

**Writing – review & editing:** Kit Moran, Emma Jones, Chad Coles, Paige Moorhouse, Andrew Glennie.
